# Training, Immunity, and Health in Elite Kayaking: A Longitudinal Study Monitoring a World-Class Marathon Paddler with Exercise-Induced Bronchoconstriction

**DOI:** 10.3390/sports13110401

**Published:** 2025-11-07

**Authors:** José Augusto Rodrigues dos Santos, Tiago Rama, Liliana Carina Baptista, Ana Isabel Padrão, Rodrigo Zacca

**Affiliations:** 1 Centre of Research, Education, Innovation and Intervention in Sport (CIFI_2_D), Faculty of Sports, University of Porto, 4200-450 Porto, Portugal; jaugusto@fade.up.pt; 2 Service of Immunoallergology, University Hospital Centre of São João, 4200-319 Porto, Portugal; tarama@med.up.pt; 3 Service of Basic and Clinical Immunology, Faculty of Medicine, University of Porto, 4200-319 Porto, Portugal; 4 Faculty of Sport Sciences and Physical Education, University of Coimbra, 3040-248 Coimbra, Portugal; lbaptista@fcdef.uc.pt; 5 Interdisciplinary Center for the Study of Human Performance (CIPER-UC), Faculty of Sport Sciences and Physical Education, University of Coimbra, 3040-248 Coimbra, Portugal; 6 Research Center in Physical Activity, Health and Leisure (CIAFEL), Faculty of Sports, University of Porto (FADEUP), 4099-002 Porto, Portugal; anaisabelpadrao@outlook.pt; 7 Laboratory for Integrative and Translational Research in Population Health (ITR), 4050-600 Porto, Portugal; 8 Nucleus of Research in Human Motricity Sciences, Universidad Adventista de Chile, Chillán 3780000, Chile; 9 Laboratory of Sport Physiology, Faculty of Sports, University of Porto, Rua Dr. Plácido da Costa 91, 4200-450 Porto, Portugal

**Keywords:** training monitoring, training and testing, training process, immune system, Kayaking, asthma, exercise-induced bronchoconstriction

## Abstract

Background: Exercise-induced bronchoconstriction (EIB) is common in athletes, being more frequent in outdoor endurance-based/long-distance sports. We followed a World-Class marathon paddler’s season with recurrent episodes of EIB, which intensified during cold exposure workouts. This unique immunophenotype profile during the season and its variations were reflected in acute and chronic inflammatory markers. Methods: A longitudinal case study was conducted with blood sampling obtained from a single paddler after overnight fasting at three timepoints: T1 (beginning of season, after 15-day rest period), T2 (post-Winter National Championship), and T3 (post-Summer National Championship). Complete blood counts and lymphocyte immunophenotyping were performed using automated hematology analysis and multiparametric flow cytometry. Results: The total numbers of leukocytes (T1: 6.3; T2: 5.0; T3: 5.5 × 10^9^/L), neutrophils (3.1; 2.5; 2.8 × 10^9^/L), and lymphocytes (2.4; 1.8; 2.2 × 10^9^/L) declined between T1 and T2, followed by a partial recovery at T3. In contrast, monocyte counts exhibited the reverse pattern (0.41; 0.62; 0.31 × 10^9^/L). The two T cell subsets (αβ and γδ) remained relatively stable, showing only minor seasonal fluctuations. CD19+ B cells, initially at very low levels, increased steadily as the season progressed (0.05; 0.07; 0.16 × 10^9^/L). During T2, the proportion of memory lymphocytes (CD45RO+) rose, while naive cells (CD45RA+) declined; this trend was subsequently inverted at M3. Although the CD4+/CD8+ ratio varied over time, it consistently stayed below the normal reference range established for healthy controls (0.50; 0.83; 0.60 for T1, T2, and T3, respectively). Conclusions: The immune assessment of the World-Class marathon paddler revealed transient immunosuppression early in the season, marked by reduced neutrophils, a low CD4+/CD8+ ratio, and diminished CD19+ lymphocytes. Over time, immune parameters showed signs of recovery, indicating a temporary imbalance that did not impair the athlete’s physical performance. Conclusions: This case study of an elite marathon kayaker revealed transient immune fluctuations across a competitive season, including early immunosuppression (low neutrophils, CD4+/CD8+ ratio 0.50, and minimal CD19+ B cells) followed by partial recovery mid- and late-season. Despite persistently inverted CD4+/CD8+ ratios suggesting chronic immune dysregulation, the athlete maintained competitive performance, highlighting the temporary nature of these changes and emphasizing that regular immune monitoring can help optimize health and performance in elite athletes.

## 1. Introduction

Recruiting high-performance athletes as volunteers for research in sports sciences and health is always a challenging task from both logistical and scheduling management perspectives [1]. In the sport of kayaking, where athletes are seated within a closed-deck boat and propel it using a double-bladed paddle, top-level competitors demonstrate performance metrics that are markedly higher than those of non-elite paddlers. This highlights the importance of distinguishing and specifically assessing this elite group to gain a clearer understanding of their distinct physiological and technical requirements [1]. Because World-Class kayakers constitute a small, technically and physiologically distinctive population [1,2], targeted case studies that provide longitudinal, individualized performance and health indicators, and contextualized analyses of training-induced adaptations are indispensable. They deliver actionable evidence that coaches, sport scientists, and clinicians need to advance training practice, injury prevention, and equipment design in competitive kayaking.

Prolonged or high-intensity training is related to a short-term suppression of immune cell activity in athletes, whereas light to moderate training tends to boost the immune defenses of this population [3,4]. The drop in immune function induced by physical training is most evident when the task is sustained, extended in duration, of moderate to vigorous intensity, and performed under fasting conditions [5]. Vigorous and extended exercise may create a brief period of reduced immune protection, often denoted as the “open window”, lasting from approximately three to seventy-two hours, during which the susceptibility to infection rises [4]. Repeated cycles of intensive training can contribute to marked reductions in immune competence. Elite and World-Class competitors normally remain asymptomatic for immune suppression. Despite that, consistent exposure to heavy training workloads can alter immune conditions and increase the vulnerability to URTIs (upper respiratory tract infections), particularly when combined with adverse environmental stressors [6,7]. In athletes with airway dysfunction such as EIB, the interplay between exercise-induced immune modulation and respiratory tract immunity becomes particularly complex, as both local airway inflammation and systemic immune responses may be simultaneously affected by training loads and environmental conditions.

Athletes frequently contend with lower airway disorders, such as asthma and EIB, during both training and competition [8]. In fact, asthma is reported to affect between 15% and 50% of Olympic-level competitors [9,10], with the prevalence of EIB reaching up to 80% in endurance disciplines [8,11,12]. In addition to symptoms originating from the smaller airways, such as chest tightness, shortness of breath, and coughing [13,14], both asthma and EIB are characterized by airway hyperresponsiveness, in which bronchial constriction is provoked by physical, chemical, or thermal stimuli [15]. Athletes with asthma usually experience ongoing airway hyperresponsiveness, with its intensity depending on the degree of airway inflammation [16]. In contrast, for those with EIB, hyperresponsiveness happens only in short episodes triggered by exertion, and it does not always relate to asthma, inflammation, or symptoms [17]. Still, when asthma or airway inflammation is not well controlled, it can make EIB more severe [18]. The sources of EIB are thought to be related to mechanical stress and airway drying [19]. Such factors elevate the osmotic pressure within the airway-lining fluid, promoting the secretion of mediators from immune effector cells (including histamine, cysteinyl leukotrienes, and prostaglandins). These substances then act on the airway smooth muscle, provoking constriction and consequent bronchial narrowing [18,19].

**Figure 1:**
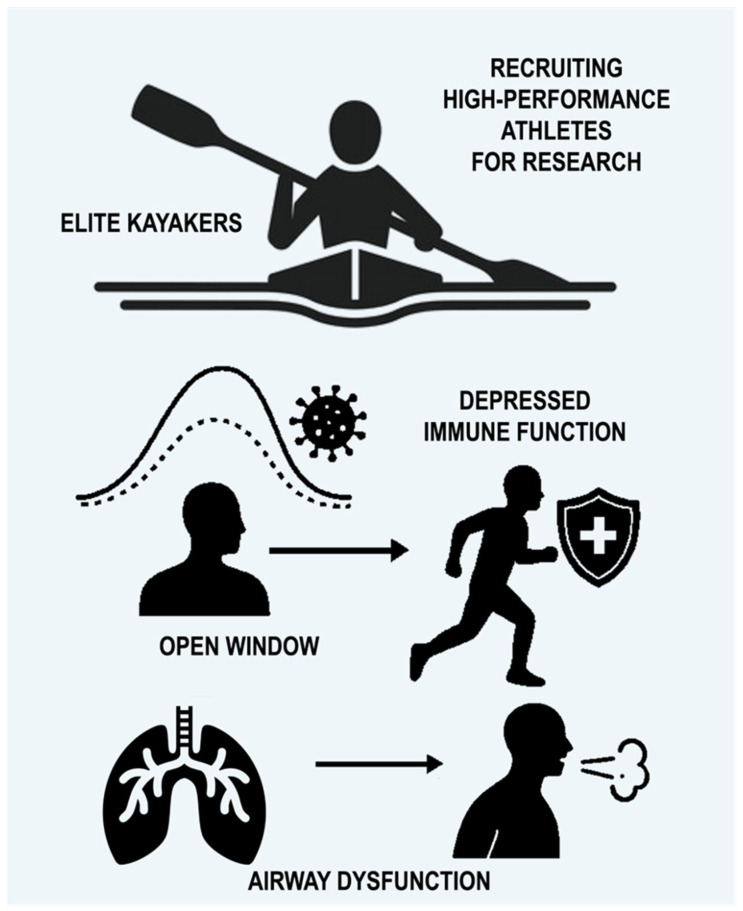
Overview of challenges in recruiting elite kayakers for research, highlighting immune function suppression during intense training (‘open window’) and the high prevalence of airway disorders like asthma and EIB in athletes. Vigorous and extended exercise may create a brief period of reduced immune protection, often denoted as the “open window”, lasting from approximately three to seventy-two hours, during which the susceptibility to infection rises [4].

The high prevalence of lower airway dysfunction in athletes is influenced by multiple factors and differs according to the type of sport and training environment [18,20]. It is well reported that substantial weekly respiratory training load seems to be an important contributor, as EIB, for example, is typically observed in endurance and ultra-endurance sports (≥6 h), irrespective of environmental triggers [18,21,22] (Figure 1).

Accordingly, we investigated immune system alterations throughout a competitive season in a World-Class marathon kayaker diagnosed with EIB.

## 2. Materials and Methods

Over a competitive season, this longitudinal case investigation evaluated the immunological characteristics of a 30-year-old World-Class male kayaker (age: 30 y) [2], a marathon specialist and member of Portugal’s national team competing in major international events, including the World and European Marathon Championships.

Data collection occurred at three stages: following a two-week recovery phase at season onset (T1); six months later, immediately after the National Winter Kayaking Championship (T2); and four months later, following the National Summer Kayaking Championship (T3).

### 2.1. Clinical Status

During the competitive season, the World-Class marathon paddler from this study presented recurrent episodes of EIB while training or competing in cold environments. Despite that, he was healthy and demonstrated outstanding physical fitness, as evaluated through both specific (kayaking) and general tests. The first assessment occurred after 8 days of complete rest following the end of the previous competitive season, which concluded with his withdrawal from the World Marathon Championship due to acute upper respiratory issues caused by a sudden drop in air temperature (35 to 15 °C) on the race day. The athlete had no previous medical management for bronchial complaints, which were exclusively triggered by vigorous physical effort. Treatment was initiated with salbutamol (100 μg, four actuations) administered twice daily, and budesonide (200 μg) prescribed for use on demand during periods of symptom worsening.

### 2.2. Training Program

The World-Class marathon paddler structured his season around two distinct performance peaks: the first culminating in the National Winter Kayaking Championship and the second concluding with the National Summer Kayaking Championship. The National Winter Kayaking Championship comprised 5000 m events in K1, K2, and K4 categories, whereas the National Summer Kayaking Championship featured a 35 km K1 marathon, typically serving as the qualifying event for international competitions such as the European and World Championships. Each training session commenced with a targeted warm-up phase consisting of calisthenic exercises prior to strength workouts or low-intensity paddling before water sessions, and lasting approximately 10 to 20 min.

**Figure 2:**
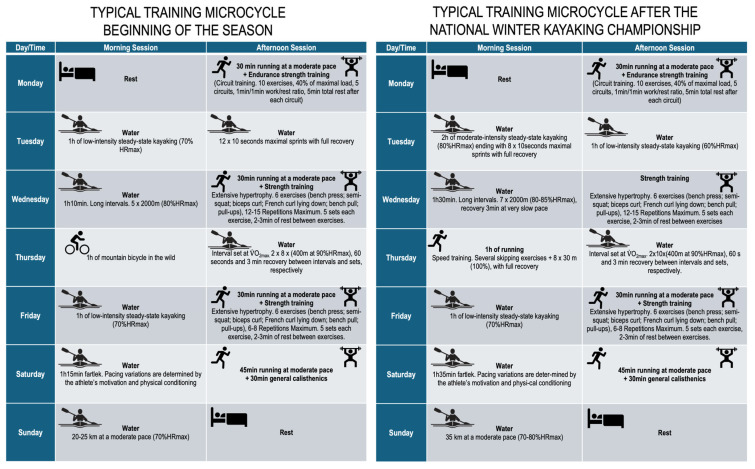
Description of the typical training microcycles.

Training intensity was monitored using heart rate (expressed as percentage of maximum heart rate of the age-predicted maximal heart rate) and athlete-reported rating of perceived exertion, though power output was not systematically recorded. The kayaking external training load variables, i.e., volume (km) and intensity (volume × strokes per minute, SPM), were ~82 km and ~4800 km × SPM, and ~168 km and 12.410 km × SPM, for the preparatory and pre-competitive periods, respectively. The description of each microcycle is detailed in Figure 2.

### 2.3. Blood Sampling

Blood samples were obtained at T2 and T3 after an overnight fast and following a 48 h period without physical training. At T1, collection occurred after eight consecutive days of complete rest. Venous blood was drawn from the antecubital vein, with 5 mL collected into tubes having K_3_EDTA as an anticoagulant. All samples were processed within 6 h of collection.

### 2.4. Analytical Procedures

Blood cell parameters were assessed using an automated hematology system (XE-5000; Sysmex Corporation, Kobe, Japan). Lymphocyte subtypes were identified through multiparametric flow cytometry (FACSCanto II; Becton Dickinson Biosciences, San Jose, CA, USA), and data interpretation was carried out with INFINICYT software (Cytognos, Salamanca, Spain). Measurements of total leukocytes and differential counts across five main cell categories were obtained following standardized analytical protocols (Max M; Coulter Electronics^®^, Hialeah, FL, USA).

### 2.5. Immunophenotyping

Mouse-derived monoclonal antibodies targeting specific leukocyte surface antigens were employed, each conjugated with either fluorescein isothiocyanate (FITC) or phycoerythrin (PE). Details regarding the antibody panels, including cluster designation, clone, fluorochrome, source, and target specificity, are provided in Table 1. All reagents were obtained from Becton Dickinson Biosciences (San Jose, CA, USA) or CT Coulter Electronics (Brea, CA, USA).

### 2.6. Flow Cytometry Samples Preparation

The lymphocyte subsets were assessed with direct immunofluorescence. EDTA-anticoagulated blood samples were incubated for 20 min at 4 °C in the dark with 15 μL of monoclonal antibodies conjugated to either FITC or PE. Red blood cells were then lysed by incubating the samples for 10 min with 2 mL of FACS Lysing Solution^®^ (Becton Dickinson, San José, CA, USA). Subsequently, the cells were rinsed with phosphate-buffered saline (PBS) and centrifuged at 1500 rpm. Within two hours of preparation, list-mode data were recorded on a FACScan flow cytometer (Becton Dickinson, San José, CA, USA).

### 2.7. Data Assessment and Analysis

We conducted flow cytometry analyses using the FACScan Lysis II research software (version 1.1; Becton Dickinson, San José, CA, USA). Lymphocyte populations were first visualized on dot plots of forward scatter (FSC) versus side scatter (SSC) and then carefully confirmed by gating on CD45 versus SSC, focusing on cells that displayed the highest CD45 fluorescence intensity. Approximately 1000 events were analyzed per sample. Photomultiplier voltages were adjusted to 600 V for FL1 and 581 V for FL2, with linear amplification applied to FL1 and spectral compensation to FL2 to ensure accurate measurements. To verify that the identified lymphocytes accurately represented the sample, their percentages were cross-checked against the distributions of the five main leukocyte subsets. Finally, gating was performed on an FL1 versus SSC plot using the CD45/CD14 Leucogate system (Becton Dickinson, San José, CA, USA). Cells showing fluorescence at least twice that of the negative control were classified as positive, ensuring reliable identification of the target population. Descriptive statistics were used. The percentage change between time points was calculated. No inferential statistics were applied due to a single-subject design.

## 3. Results

Throughout the competitive kayaking season, clear shifts were noted in the athlete’s absolute leukocyte counts. From T1 to T2, there was a noticeable drop in total leukocytes (around 20.63%), along with decreases in lymphocytes (about 25.00%) and neutrophils (roughly 19.35%). In contrast, monocyte levels rose sharply, by nearly 50% during the same period. By the time measurements were taken at T3, most of these values appeared to move back toward their initial, baseline levels. Interestingly, basophils showed a steady upward trend across the season, whereas eosinophils declined between T1 and T2 and then stabilized through T3 (Table 2).

The immunophenotype fluctuated during the season (Table 3). The CD3+, CD3+ αβ, CD3+ γδ, CD8+, and CD45RA decreased, while HLA-DR, CD4+, CD25+, CD45RO, and CD4+/CD8+, as well as CD19+, increased between T1 and T2. However, almost all indicators tended to return to initial values in T3.

## 4. Discussion

Our study set out to explore how training and competitions affect the immune profile of a World-Class marathon paddler, particularly in the context of recurring EIB. Athletes at this level, and marathon paddlers in particular, undergo long stretches of intense daily training, which can trigger noticeable acute changes in their immune systems. While exercise is a well-known trigger for bronchoconstriction in people with asthma, up to 20% of individuals without a formal asthma diagnosis can experience similar symptoms [23]. Typically, these changes are brief, returning to baseline within about 24 h [24,25]. However, chronic training can interfere with full immune recovery, prompting lasting adaptations that progressively reshape the athlete’s immune status over time. The temporal pattern of immune alterations must be interpreted within the context of training periodization. The T1 assessment followed both a severe bronchoconstriction episode and a 15-day rest period, creating confounding factors that limit baseline interpretation. The T2 assessment occurred after the winter preparation phase, characterized by high training volumes and heavy resistance training, while T3 followed a more specific competition preparation phase.

The changes observed in our current study mostly fell within standard laboratory reference ranges, which makes clinical interpretation challenging. Apart from salivary IgA, consistent and reliable markers of immunosuppression in athletes remain hard to pin down [26]. It is important to note that the first blood sample was collected just a few days after a severe EIB episode during the competition, which required oxygen support in the hospital. This condition triggered a strong mobilization of all immune systems, which was expressed by the highest values of leukocytes, neutrophils, lymphocytes, and eosinophils throughout the season. Overall, all immunological markers that shifted between T1 and T2 tended to return to their baseline levels by T3, which aligns with the findings of Bobovcak et al. [27] in elite athletes at different points throughout the season.

Low baseline leukocyte and neutrophil counts are common in athletes undergoing intense physical training, likely reflecting the movement of these cells to peripheral sites of potential exposure, such as the lungs or gut, or their role in clearing muscle debris through phagocytosis [27,28,29]. This assumption partially justifies the decrease in total leukocyte counts, mainly neutrophils and lymphocyte counts, observed in T2 and T3. From these data, it can be assumed that the highest values of leukocytes, neutrophils, and lymphocytes in T1 were a result of the preceding respiratory crisis. The decrease in lymphocytes observed in our study contrasts with the findings of Hatch-Mcchesney et al. [30], who reported the opposite trend after 22 weeks of military training.

Monocytosis is a signal that the body is acting against infection or injury. The marked increase in monocytes in T2 may be related to any subclinical infectious condition or to temporary stress from the previous strenuous workouts [31]. The recovery of initial values in T3 indicates the absence of any infectious process or better adaptation to training loads. Eosinophils play a key role in the symptoms of asthma and allergies, controlling infection. Asthmatic individuals are typically characterized by elevated eosinophil levels [32], which aligns with the high values observed at the start of this study. The decrease seen at T2 likely reflects the absence of viral or bacterial infections. Basophil counts remained within normal laboratory ranges and showed a slight increase over the course of the season.

In our study, T lymphocytes (CD3+) dropped from T1 to T2 before rising again at T3. This trend partly mirrors the observations of Baj et al. [33], who reported a significant decline in CD3+ cell counts over a cycling season. Yet, it stands in contrast to Rodrigues dos Santos et al. [34], who found these cells remained remarkably stable during a 17-day kayaking ultramarathon. Interestingly, T cell levels in our kayaker were much higher than those seen in young military recruits before and after 22 weeks of training [30].

In our observations, CD3+ αβ and CD3+ γδ cells showed only minor fluctuations, suggesting effective immune surveillance [34,35,36]. The CD3+ γδ cells, which play a wide range of immune roles, are clearly mobilized during exhaustive exercise but usually return to baseline within about 15 min [37].

In humans, about 2–4% of CD4+ cells express CD25+, identifying them as regulatory T cells that play a key role in suppressing the immune response. Our World-Class marathon paddler showed higher levels, likely related to his clinical state [32], enhanced protection against autoimmunity [38], or a heightened state of immune vigilance [39]. It seems that there is a relationship between this athlete’s intense and prolonged training model and his superior ability to fight against inflammatory processes caused by both training and his clinical condition.

HLA-DR is a molecule typically expressed by antigen-presenting cells and is associated with T cell activation [40]. Training generally does not have a major impact on immune cells expressing HLA-DR [41], which typically average around 2% (range 1–4%). In our study, however, the pronounced peak observed at T2 likely reflects the inflammatory response triggered by the preceding intense training loads, particularly heavy resistance sessions [42]. Elevated levels of HLA-DR-expressing cells can be detrimental to immune function [28] if the increase persists over time. The return to starting values in T3 seems to exclude the chronic increase in this immune indicator.

Following both moderate and heavy exercise (~50 and 80% $\dot{V}$O_2max_), NK cell counts spike dramatically but typically return to baseline within about 3.5 h [43]. Over time, however, chronic adaptation to prolonged intense training can lead to a decline in NK cell numbers [44]. In our study, the World-Class marathon paddler already exhibited high basal NK cell levels at T1, above the upper limits of laboratory reference ranges, and these values remained stable throughout the season. These levels resemble those observed in marathon runners [28], yet they contrast sharply with military trainees [30], whose basal NK cell counts (6 ± 4%) increased to 9 ± 7% after 22 weeks of training. High basal values of NK cells can express the improvement of innate defense against inflammatory processes induced by training and random attacks of bronchoconstriction during the season.

CD94+ is a cell surface molecule involved in MHC I recognition by NK cells and activated or memory CD8+ cells [45]. The proportion of CD94+–expressing cells does not appear to change in response to exercise [46] and has been shown to remain stable over time in highly trained athletes [47]. Our findings are consistent with these observations.

CD4+ cells varied marginally through the season, which is in agreement with other research in healthy active individuals [48] but diverges from the findings of Makras and colleagues, who showed a substantial increase in CD4+ after 4 weeks of intermittent moderate exercise [3], while Weiss et al. [49] showed a significant decrease in CD4+ cells after 4 weeks of anaerobic training (weight lifting and running interval training). The exercise mode and physical status of the subjects can potentially explain these discrepancies.

CD8+ T cells are the immune system’s frontline responders to antigens presented by MHC class I molecules; they proliferate, release cytokines and chemokines, and directly target infected or damaged cells [50]. To put this in perspective, a study of 273 healthy adults reported an average of ~515 CD8+ cells/μL [51]. In physically active individuals, just 30 min of cycling can push these numbers up noticeably, from ~338 to 512 cells/μL [48]. Among trained athletes, the proportion of CD8+ cells is generally higher, around 33% of total lymphocytes compared to less active peers [52]. Yet, not all studies agree, and some have reported lower values in athletes [53,54]. In our World-Class marathon paddler, CD8+ T cell counts were notably high, standing out from other reports. This makes sense: elevated CD8+ cells are crucial for managing intramuscular inflammation and supporting muscle repair [55], aligning perfectly with the demanding high-volume training that defines his regimen. On top of that, these cells play an important role in dampening airway hyperresponsiveness and reducing airway inflammation [56], which may be particularly relevant given his history of EIB [56].

The B lymphocytes might seem like “quiet sentinels” in the circulation, usually making up around 11 ± 3% of circulating lymphocytes in athletes [52]. Under normal conditions, they hold steady [50], barely changing even over the course of a full competitive season [57]. However, intense training can shake this balance; CD19+ cells sometimes dip when the body is pushed to its limits [58,59]. In our World-Class marathon paddler, this was evident at T1, where B cell counts were unusually low, far below standard reference ranges [60]. This likely reflected a tactical migration to the upper respiratory tract, where B cells face heightened immune challenges, followed by programmed cell death [61]. Over time, however, the story shifted. By T2 and T3, B cell levels gradually rebounded, perhaps aided by anti-asthma therapy or the athlete’s finely tuned adaptive immune system, slowly restoring equilibrium after the initial strain. In fact, the immune system adapts, recovers, and responds dynamically to the demands of elite performance.

The CD4+CD45RO and CD4+CD45RA T cells showed small alterations. However, memory and naive cells (%) in the CD8+ subset presented evident variations during the season. Despite that, the number and percentage of memory and naive T cells of the CD8+ subset are within the range for healthy adults [62].

Although the genetic regulation of effector and memory CD8 T cell differentiation continues to be not fully defined [63], it is now well established that the cytotoxic T lymphocytes can be divided into short-lived and memory precursor effector cells [64]. In T1, the low values of CD8+CD45RO+ T cells might be linked to the relocation of the effector cells to the sites of infection and inflammation, which induced their rapid apoptosis, while they are maintained in the long-lived CD8 T cell memory [63]. More than 90% of T cells in the primary immune response are rapidly eliminated [65]. This dramatic loss appears to serve as a key trigger to activate the long-term memory T cells, which, at least in part, support subsequent responses by naive T cells. Therefore, there may be a relationship between low levels of memory and high levels of naive T cells after the first blood collection. In quantitative terms, memory and naive T cells varied in opposite ways during the competitive season. Conflicting with our results, Woods et al. [66] showed a tendency for the percentage and number of CD4+ and CD8+ naive cells (CD45RA+) to rise and for CD4+ memory cells (CD45RO+) to reduce after 6 months of aerobic training. This inconsistency might be related to the level of training and health status of participants.

The values of CD4+/CD8+ below 0.5 are an index of high mortality risk in people living with HIV [67]. In fact, the CD4+/CD8+ ratio in healthy people is not well known but varies approximately in a 2:1 ratio [68]. Baseline values in young active subjects are usually higher than 1.5 [69]. In the opposite direction, the CD4+/CD8+ ratio < 1.0 is frequently related to some diseases. Nevertheless, morbidity and mortality rates are linked with low CD4+/CD8+ ratios [70]. The CD4+/CD8+ ratio decreases after exercise, returning to basal values within 60 min [71]. After a kayaking ultramarathon, the CD4+/CD8+ ratio decreased, increasing further during the recovery period [34], which is not consistent with the starting value of our marathoner, which is similar (0.5) to some values observed for cancer patients in their worst recovery diagnosis [72]. The trivial increase in the CD4+/CD8+ ratio shown in T2 does not mean a substantial progress in the immune response but an incidental reduction in CD8+ T cells. The persistently low CD4+/CD8+ ratio throughout the season warrants careful clinical consideration. While isolated inversions of this ratio are not uncommon in healthy athletes undergoing intense training, the consistently low values (all timepoints < 1.0, with T1 = 0.50, T2 = 0.83, T3 = 0.60), combined with the athlete’s respiratory pathology, suggest the possibility of chronic immune activation or dysregulation. However, this athlete demonstrated excellent physical performance and no clinical signs of immunodeficiency or increased infection susceptibility during the monitoring period, highlighting the complexity of interpreting immune biomarkers in elite athletes with pre-existing conditions. Signs of inflammation are a normal response to intense and prolonged physical loads and are key regulators of training adaptation [73]. However, in this athlete, normal inflammatory signs are conditioned by their clinical status.

We acknowledge some shortcomings and potential limitations in the current study. Firstly, the absence of a matched control group (either elite kayakers without EIB or individuals with EIB who are not elite athletes) prevents the determination of whether observed patterns result from training, EIB, or their interaction. However, the participation of elite athletes in control groups is limited, as they are few and often focused on competitions, which can interfere with their training. Therefore, many studies use athletes’ pre-intervention data as their own control, allowing within-subject comparisons while maintaining scientific validity. This approach eliminates interindividual differences, but the findings are specific to the evaluated athletes and may have limited generalizability to other populations. Secondly, EIB was diagnosed based on clinical history of EIB symptoms and documented bronchospasm requiring therapeutic assistance. The severity of EIB episodes was not systematically measured using standardized tests/protocols (e.g., spirometry), which represents a limitation of this study. Medication adherence was monitored through athlete self-report but not objectively verified. Finally, the test–retest reliability assessment was not applied for immunological assessments in our current case study. The inter-assay coefficient of variation for lymphocyte subset quantification usually ranges from 5 to 15% in clinical settings, and the intra-assay values are in ranges lower than 5%. Nevertheless, biological variability of immune cell counts can exceed analytical variability in World-Class athletes, with reported CV values of 15–25% for various lymphocyte subsets. We are aware that the absence of repeated measures within timepoints limits the ability to distinguish true biological changes from measurement variability when interpreting longitudinal changes in a single individual. Despite that, it is important to note that elite athletes represent a highly specific population with unique attributes. In fact, recruiting World-Class athletes for research purposes is exceptionally challenging.

### Practical Implications

While the athlete maintained outstanding physical conditioning and competitive performance, the persistent immune instability warrants closer medical monitoring, particularly regarding infection risk and EIB management optimization. Future research should examine whether these immune patterns are specific to athletes with respiratory pathology or represent more generalizable responses to elite-level training in kayaking. Prospective studies with larger cohorts, repeated measurements for reliability assessment, respiratory function testing, and immune monitoring are needed to establish evidence-based guidelines for managing elite athletes with EIB.

## 5. Conclusions

This longitudinal case study of a World-Class marathon kayaker revealed transient fluctuations in immune function across a competitive season. At the beginning of the season, the athlete showed signs of immunosuppression, including a low neutrophil count, markedly reduced CD4+/CD8+ ratio, and low values for CD19+ B cells. It remains challenging to disentangle the effects of high-intensity training from potential episodes of upper respiratory tract infections. Mid-season assessments indicated partial immune recovery, with increased CD19+ B cells, a rise in CD45RO+ memory T cells, and concurrent improvements in the CD4+/CD8+ ratio. By the end of the season, most immune indicators tended to return toward baseline values. The persistently inverted CD4+/CD8+ ratio throughout the season may reflect chronic immune dysregulation, potentially associated with the combination of intense training demands and underlying respiratory pathology. Despite these immune fluctuations, the athlete maintained competitive performance, suggesting that the observed immunological changes were temporary and did not overtly compromise physical capacity. These findings highlight the need for careful immune monitoring in elite athletes, particularly those with pre-existing respiratory conditions, and underscore the importance of considering both physiological and clinical factors when interpreting immune data.

## Figures and Tables

**Table 1:** Cluster, clone, fluorescent stain, origin, and antibody specificity.

Cluster	Main Cell Reactivity	Origin	Fluorochrome	Clone
CD3+	T cells	BD *	FITC	SK 7
CD4+	Helper/inducer T cells	CT **	PE	SK 3
CD4+CD45RA+	Effector CD4 cells	CT **	FITC/RD1	13B8.2/2H4
CD4+CD45RO	Memory CD4 cells	CT **	FITC/PE	13B8.2/UCHL1
CD4+CD25+	Activated CD4 cells	BD *	FITC/PE	SK3/2A3
CD8+	Cytotoxic/suppressor T cell	BD *	PE	SK 7
CD57+/CD8+	T cells, not restri. to MHC	BD *	FITC/PE	HNK-1, SK 1
CD3+αβ	Terminal T cells	BD *	FITC/PE	SK7/T10B9.1A-31
CD3+γδ	Cytotoxic cells	BD *	FITC/PE	SK7/B1
CD3+CD25+	Activated T cells	BD *	FITC/PE	SK7/2A3
CD3+CD16+&56+	Natural Killer cells	BD *	FITC/PE	SK 7, B 73.1, My 31
CD3+HLA-DR	Activate T cells	BD *	FITC/PE	SK/G46-6
CD8+CD45RA	Effector CD8 cells	CT **	FITC/PE	B911/2H4
CD8+CD45RO	Memory CD8 cells	CT **	FITC/PE	B911/UCHL1
CD56HLA-DR	Activated NK cells	BD *	FITC/PE	NCAM6.2/G46-6
CD94+	NK cells		FITC	HP-3D9
CD19+	B cells	BD *	FITC	4 G 7
HLA-DR	Activated cells	BD *	PE	G46-6
CD45RA	Effector cells	CT **	PE	2H4
CD45RO	Memory cells	CT **	PE	2A3
TCR γδ	Cytotoxic T cells CD4-CD8-	BD *	PE	11 F2

Legend: ** CT, Coulter Electronics, Brea, California, USA; * BD, Becton Dickinson, San José, California, USA; phycoerythrin (PE); fluorescein isothiocyanate (FITC); isotype controls of BD and Coulter were applied.

**Table 2:** Immune changes during a competitive kayaking season.

Cells	T1	T2	T3
Leukocytes (×10^9^/L)	6.30	5.00 ↓	5.50 ↑
Lymphocytes (×10^9^/L; %)	2.41 (38.25%)	1.77 (35.40%) ↓	2.23 (40.55%) ↑
Neutrophils (×10^9^/L; %)	3.10 (49.21%)	2.50 (50.00%) ↓	2.80 (50.91%) ↑
Neut/Lymp. ratio	1.28	1.41 ↑	1.25 ↓
Monocytes (×10^9^/L; %)	0.41 (6.51%)	0.62 (12.40%) ↑	0.31 (5.64%) ↓
Eosinophils (×10^9^/L; %)	0.31 (4.92%)	0.12 (2.40%) ↓	0.11 (2.00%) ↓
Basophils (×10^9^/L)	0.021 (0.33%)	0.033 (0.66%) ↑	0.041 (0.75%) ↑

Laboratory reference ranges: leukocytes, 4.00–10.00 × 10^9^/L; lymphocytes, 1.00–4.00 × 10^9^/L; neutrophils, 2.00–7.50 × 10^9^/L; monocytes, 0.20–0.80 × 10^9^/L; eosinophils, 0.00–0.50 × 10^9^/L; basophils, 0.00–0.10 × 10^9^/L. Reference change values (RCVs) for biological plus analytical variability: leukocytes, ±32%; lymphocytes, ±38%; neutrophils, ±35%. T1—Baseline values; T2—After National Winter Kayaking Championship; T3—After National Winter Kayaking Championship; ↑ increase from previous timepoint (highlighted green); ↓ decrease from previous timepoint (highlighted red).

**Table 3:** Lymphocyte subsets change during a competitive kayaking season (×10^9^/L and %).

Cells: ×10^9^/L (% of Lymphocytes)	T1	T2	T3
CD3+ (×10^9^/L; %)	1.91 (79.3%)	1.31 (74.0%) ↓	1.69 (75.8%) ↑
CD3+CD16+/56+ (×10^9^/L; %)	0.072 (3.0%)	0.012 (0.7%) ↓	0.051 (2.3%) ↑
CD3+αβ (×10^9^/L; %)	1.72 (71.4%)	1.18 (66.7%) ↓	1.55 (69.5%) ↑
CD3+γδ (×10^9^/L; %)	0.098 (4.1%)	0.047 (2.7%) ↓	0.082 (3.7%) ↑
CD3+HLA-DR (×10^9^/L; %)	0.031 (1.3%)	0.42 (23.7%) ↑	0.035 (1.6%) ↓
CD3+CD25+ (×10^9^/L; %)	0.27 (11.2%)	0.25 (14.1%) ↓	0.30 (13.5%) ↑
CD4+ (×10^9^/L; %)	0.68 (28.2%)	0.64 (36.2%) ↓	0.67 (30.0%) ↑
CD4+CD45RA (×10^9^/L; %)	0.32 (13.3%)	0.39 (22.0%) ↑	0.33 (14.8%) ↓
CD4+CD45RO (×10^9^/L; %)	0.41 (17.0%)	0.32 (18.1%) ↓	0.36 (16.1%) ↑
CD4+CD25+ (×10^9^/L; %)	0.26 (10.8%)	0.21 (11.9%) ↓	0.24 (10.8%) ↑
CD8+ (×10^9^/L; %)	1.28 (53.1%)	0.77 (43.5%) ↓	1.11 (49.8%) ↑
CD8+CD45RA (×10^9^/L; %)	1.03 (42.7%)	0.35 (19.8%) ↓	0.89 (39.9%) ↑
CD8+CD45RO (×10^9^/L; %)	0.19 (7.9%)	0.50 (28.2%) ↑	0.23 (10.3%) ↓
CD3-CD8+ (×10^9^/L; %)	0.20 (8.3%)	0.10 (11.3%) ↑	0.19 (8.5%) ↓
CD16+/56+ (×10^9^/L; %)	0.56 (23.2%)	0.37 (20.9%) ↓	0.49 (22.0%) ↑
CD19+ (×10^9^/L; %)	0.05 (2.1%)	0.07 (4.0%) ↑	0.16 (7.2%) ↑
CD25+ (×10^9^/L; %)	0.32 (13.2%)	0.29 (16.4%) ↑	0.31 (17.0%) ↑
CD56HLA-DR (×10^9^/L; %)	0.01 (0.4%)	0.046 (2.6%) ↑	0.004 (0.2%) ↓
CD94+ (×10^9^/L; %)	0.37 (15.4%)	0.37 (20.9%) →	0.31 (13.9%) ↓
CD94HLA-DR (×10^9^/L; %)	0.01 (0.4%)	0.096 (5.4%) ↑	0.02 (0.9%) ↓
HLA-DR (×10^9^/L; %)	0.07 (2.9%)	0.57 (32.2%) ↑	0.18 (8.1%) ↓
CD45RA (×10^9^/L; %)	1.73 (71.8%)	0.80 (45.2%) ↓	1.63 (73.1%) ↑
CD45RO (×10^9^/L; %)	0.65 (27.0%)	1.12 (63.3%) ↑	0.64 (28.7%) ↓
CD4+/CD8+ ratio	0.53	0.83 ↑	0.60 ↓

Laboratory reference ranges for absolute counts (×10^9^/L) in healthy adults: CD3+ 0.70–2.10; CD4+ 0.30–1.40; CD8+ 0.20–0.90; CD19+ 0.10–0.50; CD16+/56+ 0.09–0.60. CD4+/CD8+ ratio reference range: 1.00–3.60. T1—Baseline values; T2—After National Winter Kayaking Championship; T3—After National Winter Kayaking Championship; ↑ increase; ↓ decrease; → no change from previous timepoint.

## Data Availability

The data presented in this study are only available upon request from the corresponding author. The data are not publicly available as they contain information that could compromise the privacy of study’s participant.
